# Sex-Dependent Effects of Caloric Restriction on the Ageing of an Ambush Feeding Copepod

**DOI:** 10.1038/s41598-017-12661-4

**Published:** 2017-10-04

**Authors:** Enric Saiz, Albert Calbet, Kaiene Griffell

**Affiliations:** Institut de Ciències del Mar – CSIC, Pg. Marítim de la Barceloneta 37-49, 08003 Barcelona, Catalonia Spain

## Abstract

Planktonic copepods are a very successful group in marine pelagic environments, with a key role in biogeochemical cycles. Among them, the genus *Oithona* is one of the more abundant and ubiquitous. We report here on the effects of caloric (food) restriction on the ageing patterns of the copepod *Oithona davisae*. The response of *O. davisae* to caloric restriction was sex dependent: under food limitation, females have lower age-specific mortality rates and longer lifespans and reproductive periods; male mortality rates and life expectancy were not affected. Males are more active swimmers than females, and given their higher energetic demands presumably generate reactive oxygen species at higher rates. That was confirmed by starvation experiments, which showed that *O. davisae* males burn through body reserves much faster, resulting in shorter life expectancy. Compared with common, coastal calanoid copepods, the effects of caloric restriction on *O. davisae* appeared less prominent. We think this difference in the magnitude of the responses is a consequence of the distinct life-history traits associated with the genus *Oithona* (ambush feeder, egg-carrier), with much lower overall levels of metabolism and reproductive effort.

## Introduction

Copepods are a very successful group of small crustaceans that accounts for the major part of zooplankton biomass in the oceans. They play key roles in the transfer of primary production to upper trophic levels^1^ and contribute to pelagic biogeochemical fluxes, by both vertical transport and nutrient regeneration in the photic layer^2,3^. Recent studies have shown that, although most copepods are presumed to be eaten, ca. 30% of total mortality of copepods at a global scale is not from predation^4–6^. The components of this non-predatory mortality have been little studied in planktonic copepods, and they include the effects of infections and parasites, together with natural intrinsic mortality and other age-related processes^7–9^.

Ageing in animals involves shifts in the delicate balance between the generation of reactive oxygen species in cells as a result of metabolism, and the actions of antioxidant defences and cell-repair systems^10,11^. In the case of copepods, Rodríguez-Graña *et al*.^12^ made the first attempt to link oxidative damage with ageing in copepods; they examined the age-dependent changes in the levels of carbonylated proteins (a biomarker of oxidative damage to proteins) in the copepod *Acartia tonsa*. Their results were not fully conclusive, and only males appeared to show an increase in protein damage with age. Later, Saiz *et al*.^13^, studying the closely related copepod species *Paracartia grani*, found that the deterioration of vital rates and rise in mortality associated with age (true senescence) were related to an increase in oxidative damage (lipid peroxidation). This increase in oxidative damage was accompanied by a rise in the relative content of the fatty acid 22:6(n-3), an essential component of cell membranes that increases their susceptibility to peroxidation. The activity of cell-repair enzymes also increased with age, but it could not fully cope with the increase in lipid peroxidation, eventually leading to death.

Age-related processes in copepods do not only have an influence on individual performance, but can also affect the dynamics of populations and have implications at the community and ecosystem levels^14–16^. Both female fecundity and male mating capacity decrease with age in copepods^15,17–19^; and reductions in the lifespans of females and males have been linked to the cost of mating^15,17,20,21^. Moreover, the sex-specific lifespans and ageing of several copepod species have been linked to the trade-offs associated with species-specific behavioural patterns (feeding, mate-finding, spawning)^14^.

The trade-offs between survival (somatic maintenance) and reproduction can be multifaceted, and often the causal mechanisms of reproductive costs are not well known^14,22,23^. Production of eggs is energetically demanding, and, consistent with life-history theory, high investment in reproduction causes a reduction in the life expectancy of animals^22^, including copepods^15,17,20,21^. However, greater access to food is not necessarily invested in living longer, but may be used to obtain a higher reproductive output over a shorter time span^23^. In this regard, it has been long known that the lifespans of a wide range of animals can be extended in the laboratory by restricting their food intake (termed *caloric restriction*)^24,25^. The underlying mechanisms relating caloric restriction and lifespan are not yet fully understood, but seem to be related to overall attenuation of oxidative stress and enhancement of defences against oxidative damage^24,25^. Saiz *et al*.^13^ reported the first evidence of the effects of caloric restriction in marine copepods. As in many other animal models, caloric (food) restriction lowered age-specific mortality rates and increased survival times of the calanoid copepod *P. grani*. The increase in its life expectancy under food restriction was associated with decreased reproductive effort. It is still uncertain, however, whether the findings of Saiz *et al*.^13^ can be generalized to planktonic copepods with markedly different life history traits, and for which reproductive and somatic costs may have a different balance.

We report here results from a study of the effects of caloric restriction on the marine cyclopoid copepod *Oithona davisae*. The genus *Oithona* is considered to be the most widely distributed and abundant copepod genus in the oceans. The species belonging to it are generally small and have low fecundities associated with their sac-spawning reproduction^26,27^. They are strict ambush predators, mostly motionless while relying on mechanoreceptors in the antenna to detect passing motile prey^28–30^. As a consequence of these markedly distinct behavioural and reproductive strategies, *Oithona* spp. are typically considered to be copepods with low activity and low metabolism^31,32^. Our hypothesis is that these differential traits would affect the outcome of exposing *O. davisae* to caloric restriction, being overall less sensitive to it. Moreover, because the males of this species are active and fast swimmers when searching for females, they present a shorter life span^15,33^. Thus, we also postulate the existence of distinct, sex-dependent ageing responses to caloric restriction.

In order to test these hypotheses, we have measured the life expectancy of male and female *O. davisae* under food-restricted and food-replete conditions. We have also estimated the age-dependent recruitment rate and the total reproductive effort of females throughout their lifespan under both food treatments. For a better interpretation of our results, we have also assessed the tolerance to starvation of adult male and female *O. davisae* and the duration of female reproduction without food.

## Results

### Effects of caloric restriction on the survival of adult males and females of *Oithona davisae*

**Figure 1:**
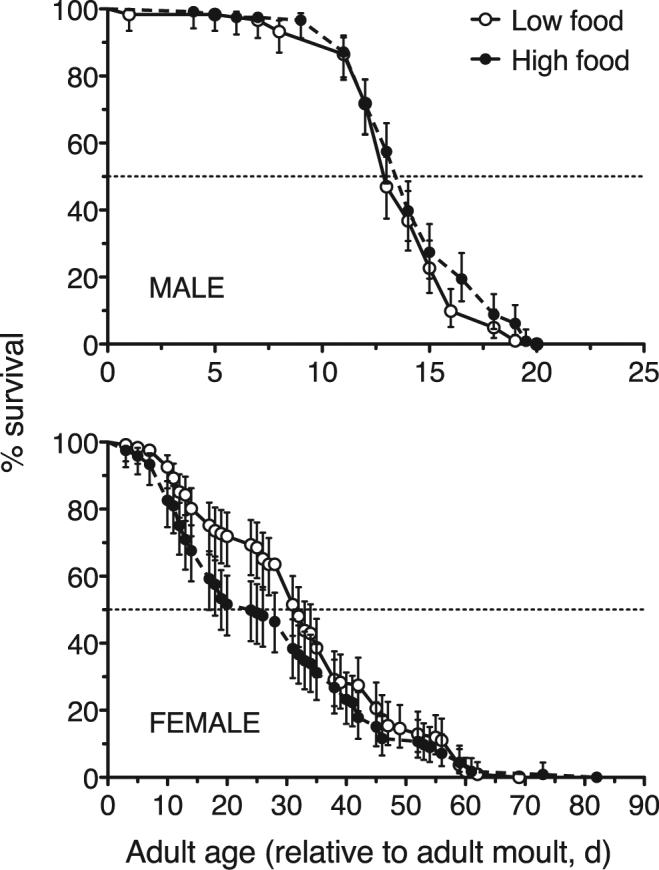
Survival in the caloric restriction experiment. Kaplan-Meier survival curves for adult males and females of the copepod *O. davisae*. Low and high food conditions correspond to, respectively, caloric restriction and satiation. Error bars are ±95% confidence interval. Dotted line illustrates the 50% of the population.

We assessed the survival of males and females exposed to high and low food rations by monitoring individual females from the time they entered the adult stage until death. Overall, males of *O. davisae* lived much shorter lives than females, with median survival times of 13 (95% CI: 13–14) and 14 (95% CI: 13–14) days when exposed to low and high food availability, respectively (Fig. 1). Mean life expectancies (±s.e.m.) of males at low and high food availability were, respectively, 13.6 ± 0.28 and 14.2 ± 0.28 days. No significant differences were found in the survival curves of males in the caloric-restriction experiment (Fig. 1; Log-rank (Mantel-Cox) and Gehan-Breslow-Wilcoxon tests, p = 0.084 and p = 0.245, respectively).

For adult females, we found median survival times of 32 (95% CI: 31–35) and 24 (95% CI: 17–31) days for the copepods exposed to low and high food availability, respectively (Fig. 1); mean life expectancies (±s.e.m.) were 32.5 ± 1.44 and 27.6 ± 1.59 days, respectively. The Gehan-Breslow-Wilcoxon test showed the differences in female survival curves were statistically significant (p = 0.012), but the probability estimated by the Log-rang (Mantel-Cox) test was higher (p = 0.114). This latter test gives more weight to a few extreme extensions of the life span; the Gehan-Breslow-Wilcoxon test gives more weight to deaths at early time points and, therefore, was more sensitive to the evident differences in the survival curve from our experiment (differences more apparent at female ages between 15 and 30 days, Fig. 1).

**Figure 2:**
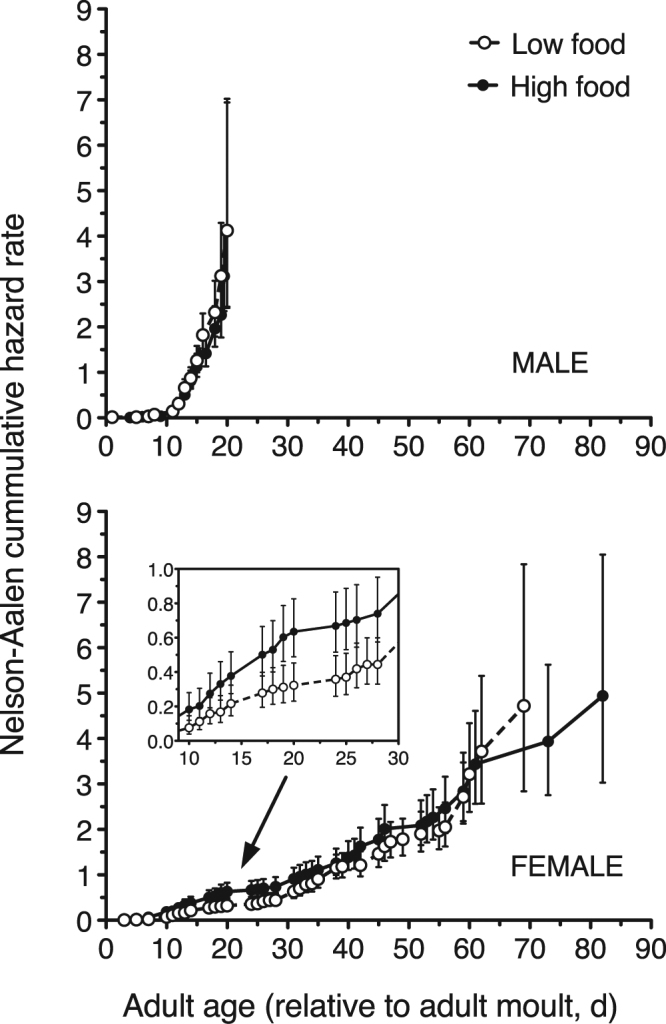
Mortality in the caloric restriction experiment. Nelson-Aalen cumulative hazard rate for adult males and females of the copepod *O. davisae*. Low and high food conditions correspond to, respectively, caloric restriction and satiation. Female plot also shows an inset enlargement of the hazard rates between days 10 and 30. Error bars are ±95% confidence interval.

The Nelson-Aalen cumulative hazard (mortality) rates for male and female *O. davisae* in the caloric-restriction experiment indicate that age-specific mortality rates increased with age; the cumulative hazard increased much faster in males than in females (Fig. 2). Regarding the effect of food restriction, the cumulative hazard rate curves showed the same pattern as observed with the survival curves: complete overlap of curves in the case of males, and higher hazard rates for females fed the high food concentrations than the low ones in the age interval between ca. 15 and 30 days (notice that 95% confidence intervals do not overlap; Fig. 2).

### Effects of caloric restriction on the fecundity of adult female *Oithona davisae*

**Figure 3:**
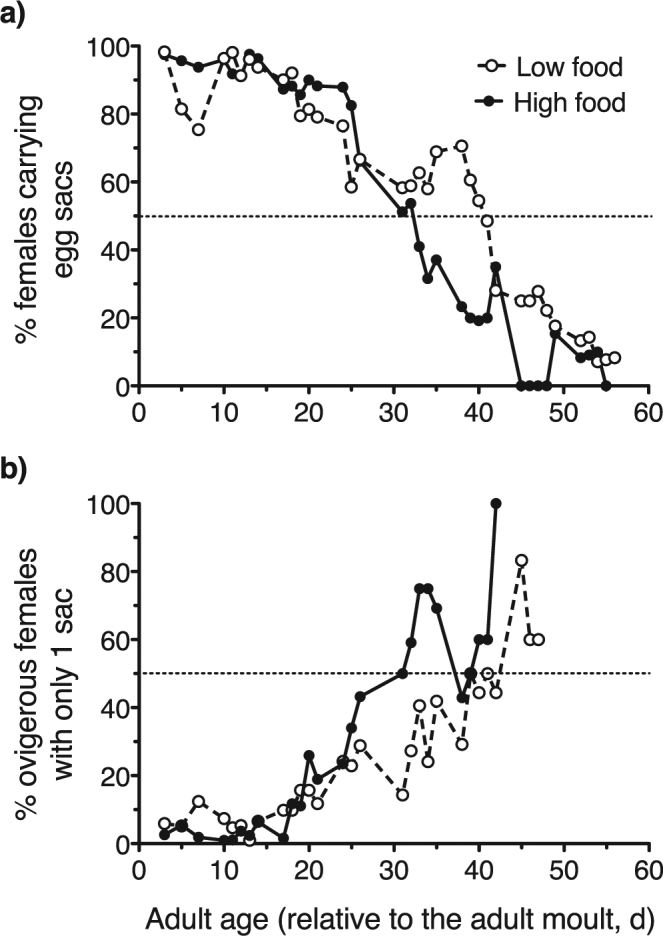
Reproductive status of *O. davisae* females in the caloric restriction experiment. (**a**) Time course of the relative abundance of ovigerous females (i.e. carrying egg sacs; as percentage of the total female population) through the adult cohort span. (**b**) Time course of the relative abundance of females carrying only 1 egg sac (as percentage of the number of ovigerous females) through the adult cohort span. Low and high food conditions correspond to, respectively, caloric restriction and satiation. Dotted line illustrates the 50% of the population.

The percentage of ovigerous females (i.e. carrying either 1 or 2 egg sacs) declined with age, and it dropped faster in the high food treatment, as measured by threshold at 50% of females ovigerous (Fig. 3a). This threshold was reached around day 33 at high food concentration, whereas at the low food concentration it was reached around day 41 (Fig. 3a). At the beginning of the monitoring most of the females carried two egg sacs, but after the first two weeks the proportion of females carrying only one sac gradually increased, faster for the females kept at high food concentration (Fig. 3b); this decline in paired-egg frequency was possibly due to the senescence of the ovaries that eventually went inactive.

**Figure 4:**
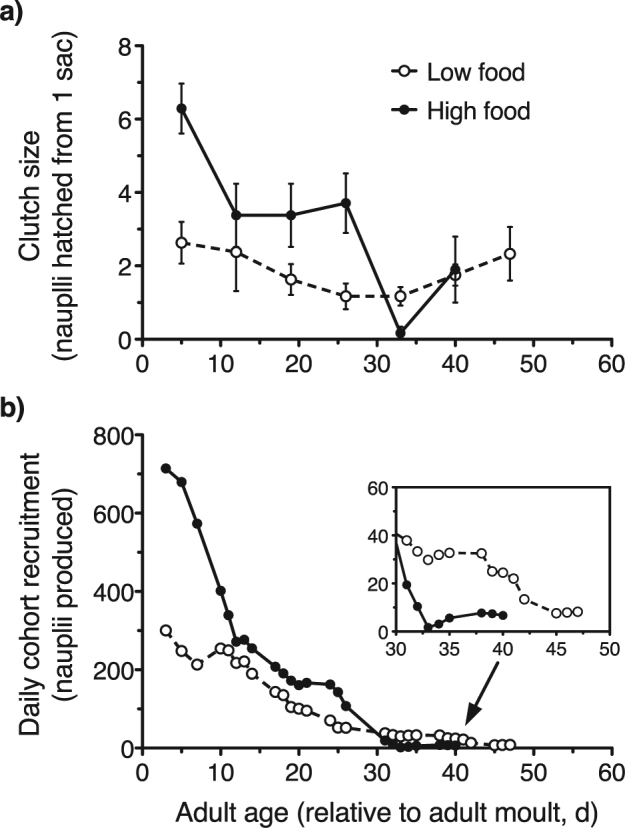
Nauplius production by *O. davisae* females in the caloric restriction experiment. (**a**) Clutch size of females (assessed as nauplii hatched per sac in 48 h) versus age. (**b**) Time course of the daily cohort recruitment (as number of nauplii produced by the live females present in the cohort). Low and high food conditions correspond to, respectively, caloric restriction and satiation. Error bars are ±s.e.m.

The changes in the clutch size of *O. davisae* females as the caloric-restriction experiment progressed are shown in Fig. 4a. Under high food concentration nauplius production was high at the beginning (ca. 6.2 nauplii hatched per egg sac in 48 h), and then decreased but remained stable for about three weeks (ca. 3.5 nauplii hatched per egg sac in 48 h). After that, production rates dropped. At low food concentration, nauplius production rates were lower, but were rather steady for most of the female reproductive period. The rise in rates observed for the longest lived of the cohorts was likely a response to the low abundance of females at that time (only 5 ovigerous females remaining in each treatment). The mean (±s.e.m.) daily fecundities during the first week, respectively 2.6 ± 0.57 and 6.3 ± 0.68 nauplii female^−1^ d^−1^ in the low and high food treatments, agree with the values reported for *O. davisae* at similarly food-restricted and satiating concentrations^27^. Those earlier results confirm that the copepods in our experiments were really experiencing food-restricted and food-satiating conditions.

Throughout their life span *O. davisae* females can produce averages of 59 and 109 nauplii at low and high food concentration, respectively; most of these nauplii (respectively 50 and 98 nauplii) were produced during the age intervals up to the median survival times (low food: 32 days; high food: 24 days). Figure 4b shows the time courses of nauplius recruitment from the female cohorts through their life spans, calculated from the age-dependent numbers of ovigerous females in the cohort and the weekly determinations of nauplius production rates (clutch size). Under high food conditions, the accumulated total number of nauplii produced by the cohort before all were dead was 10,023 nauplii (cohort size: 118 females), whereas that was halved at low food concentration (5,441 nauplii; cohort size: 120 females). In the low food treatment, the nauplius recruitment by the cohort extended longer into the final part of the life span and showed higher levels at those ages than were exhibited by the females given high food availability (Fig. 4b).

### Sex differences in survival under starving conditions

**Figure 5:**
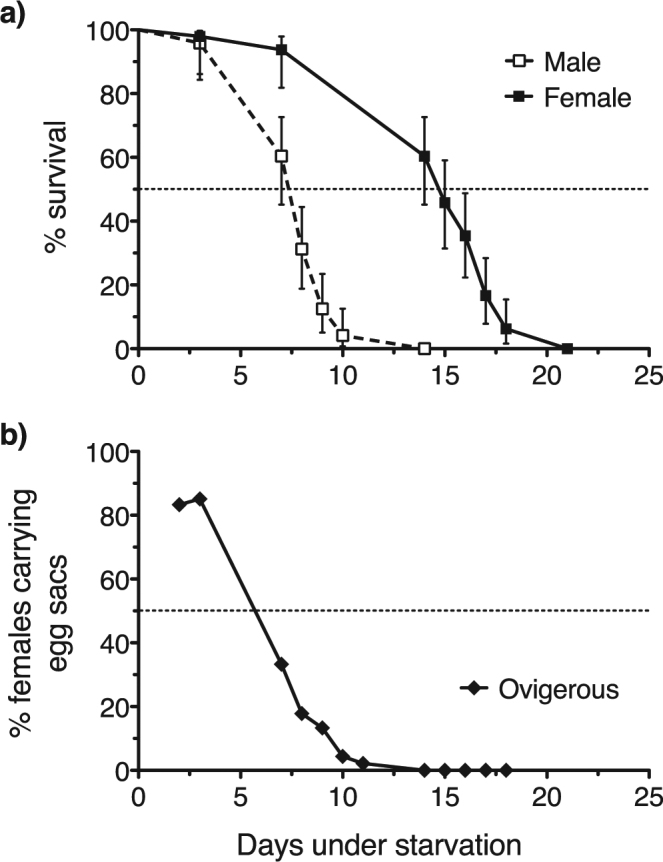
Tolerance of *O. davisae* to starvation. (**a**) Kaplan-Meier survival curves for recently moulted adult males and females subjected to starvation. (**b**) Time course of the reproductive status of recently moulted adult females subjected to starvation, expressed as relative abundance of ovigerous females with respect to total female population. Error bars are ± 95% confidence interval. Dotted line illustrates the 50% of the population.

Survival curves of male and female *O. davisae* under starvation differed markedly and significantly (Fig. 5a; Log-rank (Mantel-Cox) and Gehan-Breslow-Wilcoxon tests, both p < 0.001). The drop in survival was abrupt for both sexes. Median survival time of males incubated in filtered seawater was 8 (95% CI: 7–8) days, whereas for females it was 15 (95% CI: 14–16) days. The mean life expectancies (±s.e.m.) of starving male and female *O. davisae* were, respectively, 8.0 ± 0.27 and 15.3 ± 0.46 days (as adults).

We also quantified the production of egg sacs by starved female *O. davisae* through their adulthood until death (Fig. 5b). The spawning period under starvation ended well before the bulk of the female population died (Fig. 5a). In the absence of food, the body reserves fuelled the production of eggs until around day 11; by day 6, however, the percentage of ovigerous females was already reduced to about half of the initial percentage (ca. 84%; Fig. 5b).

## Discussion

Despite the relevance of copepods in the biogeochemical fluxes of the oceans, the biology and ecology of copepod ageing has been neglected until recently. Ageing in copepods involves age-related declines of vital rates, with an associated increase in mortality rates^12,13,19,34^. Senescence of physiological rates is accompanied by a gradual impairment of locomotive capacity that may result in greater predation risk; at the same time, senescence may also alter the chances of suffering from disease and infection. The age structure resulting from senescence, therefore, will influence the success of recruitment of copepod populations and eventually affect the dynamics of fish populations relying on them.

Life-history traits related to feeding behaviour (i.e., ambush feeder vs. active feeder) and spawning mode (i.e., broadcaster vs. egg sac-carrying) have been proposed recently as modulators of ageing patterns in copepods^14^. Our results suggest that ageing in *O. davisae* involves true senescence, as evidenced by an age-dependent increase in mortality rates in both males and females. Males showed a much faster increase in mortality, resulting in shorter lifespans (ca. 50%) in comparison with females. The shorter lifespan of males has been described previously for this species^15^, and that is not uncommon for copepods, although reported differences in the lifespans of males and females are often smaller^14,35,36^.

The much shorter lifespans of male *O. davisae* in comparison with the females must be related to the distinct life history traits of the sexes of this species. Females of *O. davisae* are strict ambush feeders, spending most of their time motionless in the water column, slowly sinking. They have low physiological rates. Males, although also ambush feeders, allocate a variable amount of time searching for females, swimming very fast along convoluted spiralling paths^37,38^. The much greater swimming activity of *O. davisae* males implies more active metabolism, and more oxidative stress, which could explain their shorter lifespans. Similarly, Rodríguez-Graña^12^ reported higher accumulation of oxidative protein damage in males than females of the copepod *Acartia tonsa*, and related that to their shorter lifespans. Paradoxically, despite the substantially greater swimming activity of males of *O. davisae*, their reported weight-specific respiration rates do not appear to be substantially different from those of females^39^. However, maximum weight-specific ingestion rates of males are similar to those displayed by the females (both at 70–80% d^−1^)^27,33^, suggesting that metabolism might be unsupported by nutrition and that exhaustion of body reserves underlies their earlier deaths. Our starvation experiments seem to confirm this, since median survival times of starved males compared to females were halved, indicating a much faster burn out of reserves. Most likely, as a consequence of this high degree of activity, males of *O. davisae* are also subject to greater oxidative damage than females, as reported for *A. tonsa* ^12^, eventually leading to their shorter lifespan.

We must notice that, compared to our data, the median survival times estimated by Ceballos & Kiørboe^15^ for the same species, *O. davisae*, were much longer overall. However, when we limit the comparison of our data to only one of their treatments, i.e. previously mated males and females incubated singly, their median survival times (respectively ca. 17 and 31 d) resemble our estimates more closely. Our experimental set-up was similar to that of Ceballos & Kiørboe^15^, since both sexes were kept isolated from each other during monitoring, but in both studies they had an opportunity to mate during the hours between moulting to the adult stage and being picked out for the experiment. Further experimentation and analysis are required to reveal the underlying mechanisms that explain the much longer lifespans reported in the other treatments in Ceballos & Kiørboe^15^.

Copepod feeding and production rates in the oceans are generally far from the potential maximum values, and copepods are commonly food limited^40,41^. Different life strategies in marine copepods have evolved through an evolutionary arms race to cope with these unfavourable periods (e.g., large lipid reserves and seasonal migration, resting eggs). It is not unreasonable to think that resource availability may also influence the patterns of senescence and mortality in copepod populations through the interaction with physiological, environmental and behavioural constraints associated with life history trade-offs^13,42^. Food (caloric) restriction strategies surely have evolved in copepods, as in other animals, to ensure survival through periods of resource scarcity, enhancing the chances for survival and recruitment of young^13,23^.

Caloric restriction is accepted now as a nearly universal paradigm; it extends lifespan and reduces age-related chronic diseases in a variety of animals^23,24^. The underlying mechanisms that link a reduction in food intake with an attenuation of the effects of oxidative stress, with a consequent reduction in the intrinsic rate of ageing, are probably not as simple as formerly thought and are still not fully understood^25^. In our experiments, we observed that the effects of caloric restriction in the ageing pattern of *O. davisae* differed between males and females. Female *O. davisae* under caloric restriction, similarly to what has been observed for females of the copepod *P. grani* ^13^ and a wide range of animals^24^, reduced age-specific mortality rates and extended the median survival time and life expectancy in comparison with those under abundant food. However, we did not observe any significant effects of food restriction (distinct from starvation) on longevity of male *O. davisae*. We think that the life history contrasts between male and female *O. davisae* (life expectancy, behaviour, metabolism) are responsible for the differential effects of caloric restriction we observed. The comparatively shorter lifespans of *O. davisae* males under starvation (ca. 50% that of the female), despite them being of relatively similar sizes, suggests much higher rates of metabolic expenditure and consequent oxidative damage.

Given their tolerance to starvation (median life expectancy: 8 d), the mean life expectancy of *O. davisae* males with food available is comparatively short. In fact, ca. 59% of the mean life expectancy of males with food (13.6 and 14.2 days at low and high food availability, respectively) could be supported by just their body reserves in the absence of feeding (mean life expectancy under starvation: 8 days). Contrarily to females, there was no substantial difference in male lifespan when exposed to caloric restriction. We speculate that the variation in food intake accomplished by *O. davisae* males under caloric restriction was probably narrow compared to the overall somatic costs of males, and therefore it did not affect significantly male lifespan. *Oithona davisae* males have low overall rates of spermatophore production (an average of 11 spermatophores over the full lifespan)^15^, but there are no estimates of the actual costs of sperm production in copepods, and it is uncertain whether or not that could be affected by caloric restriction.

In our experiments, caloric restriction of female *O. davisae* extended the female reproductive periods as well as their lifespans. Contrary to the short reproductive periods of females previously reported for *O. davisae* and *Temora longicornis* ^15,19^, we found that females in both treatments were actively reproducing almost until the ends of their lifespan. For instance, one week after the mean life expectancy, 50% of the remaining females were still reproducing. Compared to those in food-restricted treatment, the productivity of females in the high food treatment substantially declined earlier in their lifespans. Our findings here of a short post-reproductive lifespan in copepods are in agreement with reports on other species, like *P. grani* ^13^, *A. tonsa* ^12,18^, *Centropages typicus* ^9,34^ and *Eurytemora affinis* ^21,43^. For animals like copepods, with no parental care or social defence benefits, it would be difficult to explain the selective value of a prolonged post-reproductive lifespan^44,45^, and most likely in nature it would not often materialize under high predation pressure^4^.

The median survival times observed here for *Oithona davisae* at satiation (median: 24 d; 95% CI: 17–31) are not sensibly different from those reported for *P. grani* at similar conditions (median: 16 d; 95% CI: 12–30)^13^. However, compared to female *P. grani* ^13^, the response to caloric restriction in female *O. davisae* was moderate. Specifically, median survival times of female *P. grani* under food restriction were extended by factors of 2 to 4 (depending on conditions), whereas food restriction of *O. davisae* females only prolonged median survival times by a factor of 1.3. Moreover, and in agreement with life history theory, which predicts that high investment in reproduction reduces the chances of survival in many animals^33^, Saiz *et al*.^13^ found a coupling between survival and reproductive costs in their caloric-restriction experiments with copepods. Thus, they reported that the increase in life expectancy of *P. grani* under food restriction (by a factor of 2.4) was similar to the observed decrease in the lifespan-accumulated reproductive effort under the same food conditions^13^. We did not find such tight coupling in *O. davisae*: at food restriction, lifespan-accumulated reproductive effort was almost halved, whereas (as mentioned above) the increase in median survival time was moderate. We think this differential response to caloric restriction, aside from possible differences in the actual level of food limitation between the studies of the two species, is mainly a consequence of the different life history traits (locomotive activity, feeding and reproductive strategies) of these two copepod species. *Paracartia grani* females are free-spawning, active, suspension feeders, with comparatively much higher behavioural activity than the egg-carrying, ambush-feeding *O. davisae* females. For instance, reported average swimming speeds of *P. grani* females are ca. 6 times faster than those of *O. davisae* females^33^. Maximum weight-specific egg production rates of *O. davisae* at 18–20 °C are on the order of 12–20% d^−1^ ^27,46^, whereas the rates reported for *P. grani* at similar temperatures are ca. 45–63% d^−1^ ^47,48^. Moreover, at 19 °C a single *P. grani* female can spawn, under food-satiated conditions, around 2500 eggs over its lifespan^13^, which accounts for a turnover of ca. 13 times the female biomass; in comparison, in our experiments at satiating conditions *O. davisae* females spawned only 109 eggs on average over their lifespans, accounting for ca. 2.7 times the *O. davisae* carbon content. In our opinion, the comparatively lesser effects of caloric restriction we found for *O. davisae* females must be a consequence of such different degrees of biological activity between these two species. According to the free radical or oxidative stress theory of ageing, the overall low lifespan-accumulated reproductive effort of the *O. davisae* females, coupled with lower metabolic activity and maintenance costs related to their ambush behaviour, presumably results in less production of reactive oxygen species at the cellular level, allowing a much narrower range of variation of the response to caloric restriction^10,49^.

The implications of our study for the population dynamics of *Oithona davisae* and other marine cyclopoid species are several. The dynamics of this perennial species, present year around, has been described in detail in two of their habitats, the Inland Sea or Japan and the Black Sea, and appears quite consistent^46,50^. During winter (temperatures in the range 6–9 °C) practically only adult females can be found in the water column^46,50^. These overwintering females appear to be already fertilized and with functional ovaries throughout the unfavourable period, giving birth to a new generation when favourable spring conditions are reached^46,50,51^ a single copulation is enough to fertilize all eggs laid through lifespan in Oithonids^15,52^. Males do not appear in the population after the new generation has developed, and are overall scarce; even during the phase of population growth the mean sex ratios (male/female) are biased towards females (0.16 to 0.24), although sporadic episodes of male presence, with sex ratios up to 0.49, can be found^46,50^. The long overwintering period experienced by female *Oithona davisae* in the field is in agreement with our observations in the laboratory. The low winter temperatures at both locations obviously contribute to slow down the metabolism and enhance survival of females until recruitment^50^. According to our study, however, the presumably low food availability during that period will also contribute to extend both the lifespan (ca. 33% increase in median survival times) and the reproductive period of *Oithona davisae* females (Figs 3 and 4) until the next favourable season. The short life-span of male *Oithona davisae*, as shown in our study and by Ceballos & Kiørboe^15^, together with their limited mating capacity^15,38^ and biased population sex ratios^46,50^ have been proposed as causes of severe fertilization limitation in this species^15,38^; in our study, caloric restriction effects on males proved not relevant and therefore are expected to have little incidence in the field.

As concluding remark, our study confirms that the ageing response to caloric restriction of marine planktonic copepods depends on interspecific and sex-dependent differences in life history traits. Studies of more species, deeper knowledge of the trade-offs involved between selected traits and longevity, and evaluation of the proximate causes of the caloric-restriction responses of copepods are needed before a more robust and general interpretive framework can be established and applied to population dynamics^11^. Although not easy, field determinations of copepod age and of the degree of incidence of infections and/or disease^8,53^, likely borrowing from techniques used in other animal models^54,55^, are needed in the near future to assess the real relevance of senescence and age-structure effects in natural copepod populations^16^.

## Materials and Methods

### Experimental organism

*Oithona davisae* is a small marine cyplopoid copepod, commonly assumed as a model for experimental research on Oithonids^30,37,56,57^. Copepods used in this study came from the culture of the planktonic cyclopoid copepod *O. davisae* kept continuously in our laboratory since their isolation from Barcelona harbour in October 2000^58^. They are grown at 19 °C and fed the heterotrophic dinoflagellate *Oxyrrhis marina* (that are grown on the alga *Rhodomonas salina)*. Adult male and female *O. davisae* are similarly sized, although males are slightly smaller (cephalothorax length, mean ± s.e.m.; male: 345 **±** 1.3 µm, n = 31; female: 350 ± 1.6 µm, n = 39; significantly different, two-tailed t-test, P = 0.015). The dinoflagellate *Ox. marina* and the cryptophyte *R. salina* are widely used in copepod culturing and considered high quality food for copepods.

### Caloric-restriction effects on adult male and females

In order to assess the effects of caloric restriction, we followed the survival and reproductive behaviour of adult male and female *O. davisae* under limiting and satiating food conditions from the terminal moult until population death. To do that, we first generated a cohort of *O. davisae* from our continuous culture following the procedures in Saiz *et al*.^56^, and kept it at satiating conditions (>800 cells mL^−1^ of *Ox. marina*).

The cohort was periodically inspected, and when mid-stage copepodites appeared, a sufficient subsample was taken and placed in a 2-L Pyrex screw-cap bottle filled with a satiating concentration of *Ox. marina*. Regularly, the contents of the 2-L bottle were gently sieved (>120 µm) and fully inspected with a stereomicroscope, until the appearance of the first adult males and (ovigerous) females – the latter very scarce, which were all discarded from the culture. The cohort was then checked daily, and when newly moulted adult males and newly moulted females were recognizable in the culture, groups of either two adult males or two adult ovigerous females were pipetted using the stereomicroscope into the wells of multi-well plates filled with 10 mL of one of the experimental diets. Males and females were not incubated together in the wells, but they had had the chance to mate in the time previous to sorting, and females produced nauplii at expected rates (see Results section). In total, four groups, each in ten 6-well plates, were set up: males with low food, males with high food, females with low food and females with high food. Temperature in the incubation room averaged 18.7 °C (±0.29 s.d.); the light regime was set to 10 L:14D. Age 0 of a newly moulted adult *O. davisae* was defined as the day it was placed in the plates.

The limiting (“low”) and satiating (“high”) food conditions for *O. davisae* adults were set at 200 cells mL^−1^ (ca. 90 µg C L^−1^) and 800 cells mL^−1^ (ca. 370 µg C L^−1^), respectively^27,33^. Food was replaced 3 times per week (Monday, Wednesday, Friday), following the procedure in Saiz *et al*.^13^. We gently mixed the well contents and pipetted out ca. 8 mL, replacing those with fresh *Ox. marina* suspension. During the procedure, hatched nauplii were removed. The *Ox. marina* stock culture was not fed in the ca. 24 h prior to use for feeding, in order to ensure that the dinoflagellates had depleted their own food (*R. salina)*, so that only *Ox. marina* was offered as prey to the copepods. A Coulter Multisizer III particle counter fitted with a 100-µm tube was used for the preparation of the food suspensions. From previous data, and given that food was replenished every two or three days, we expected the food rations at the low and high food treatments would be, respectively, rather limiting (but not inducing starvation) and satiating or close to it^27,33^. The data on nauplius production (see Results) confirmed this assumption when compared with the egg production functional response for this species^27^. Over the weekend between day 9 and 11 of adulthood, the food concentration in the “low food” treatment was set by mistake to satiating levels. On day 12 (Monday) it was again changed to the corresponding low food concentration. We did not see any substantial effect on the biological variables measured afterwards (see Results).

The condition of the adults (dead/alive; ovigerous/non-ovigerous; 1-sac/2-sac) was monitored by visual inspection of the plates under the microscope, usually conducted during all weekdays. When dead animals were found, they were removed from the wells. Inactive individuals were carefully touched with the tip of a pipette to verify whether or not they were still alive. If any appendage moved, even if the individual showed markedly poor condition, it was considered alive. On the plates with limiting food conditions, to make food availability more homogenous throughout the experiment, wells occupied with single individuals due to the death of their partner were combined, if possible. On four occasions during the experiment, all animals were regrouped in pairs and transferred to new plates to avoid potential problems from fouling.

Kaplan-Meier survival curves, median survival times, the Nelson-Aalen cumulative hazard functions (age-specific mortality rates) and statistical tests were calculated with Prism 5.0 f. d and STATA/SE 12.1. The mean life expectancy of adults, defined as the average number of days to live after the onset of adulthood, was calculated from the area under the survival curve.

### Nauplius production experiments

Every week we conducted 48-h incubations to assess the changes in fecundity (nauplius production) through their adult lifespans. Six pairs of ovigerous females from each experimental condition were transferred to new 6-well plates with fresh suspensions of the corresponding food concentrations, and those plates were incubated for 48 h to allow the eggs to hatch. This time was sufficient to ensure hatching, because the estimated embryonic development time for *O. davisae* at our experimental temperature is 41 h^59^. The females were then returned to their original plates with the remaining females, and the contents of the “fecundity” wells were preserved with Lugol’s for counting. Nauplius production per egg sac was estimated as the ratio of nauplii found per well divided by the initial number of egg sacs carried by the two females in each well. By the end of the monitoring period the number of females incubated had to be reduced, because not enough ovigerous females were present.

The accumulated fecundities of an “average” female (i.e. up to the mean life expectancy) and of a full-lifespan female were calculated, taking into account the average number of egg sacs per (live) female and the nauplius production per sac at each time interval. Population-based estimates of recruitment were calculated taking into account the time course of mortality in the cohort and the average nauplius production for each time interval.

### Survival under starvation experiments

Additionally, we conducted an independent experiment to determine the survival time of recently moulted males and females of *O. davisae* under starvation. The general procedure was like that described above for the caloric-restriction experiments. The major difference was that males and females were monitored in filtered seawater, and only four 6-well plates were set for each sex (i.e. 48 males and 48 females; each sex incubated separately, in pairs). Age 0 of adult *O. davisae* was defined as when the survival monitoring was initiated; in this case males and females in the cohort had moulted into adulthood over the weekend (not monitored), therefore, they were actually up to 2 days old when the survival experiment started. Hatched nauplii were removed from the wells and fresh, filtered seawater replaced occasionally.

### Data availability

The datasets presented in this study are available from the corresponding author on reasonable request.
