# Publisher Correction: Sex-Dependent Effects of Caloric Restriction on the Ageing of an Ambush Feeding Copepod

**DOI:** 10.1038/s41598-017-16206-7

**Published:** 2017-11-22

**Authors:** Enric Saiz, Albert Calbet, Kaiene Griffell

**Affiliations:** Institut de Ciències del Mar – CSIC, Pg. Marítim de la Barceloneta 37-49, 08003 Barcelona, Catalonia Spain

*Scientific Reports* **7**:12662; doi:10.1038/s41598-017-12661-4; Article published online 04 October 2017

The original HTML version of this Article contained an error in the title of the paper, where the title “Sex-Dependent Effects of Caloric Restriction on the Ageing of an Ambush Feeding Copepod” was incorrectly given as “comment = “please make the changes marked up in the attached pdf” Sex-Dependent Effects of Caloric Restriction on the Ageing of an Ambush Feeding Copepod”. This has now been corrected in the HTML version of the Article. The PDF version was correct from the time of publication.

